# Volumetric imaging and morphometric analysis of breast tumor angiogenesis using a new contrast-free ultrasound technique: a feasibility study

**DOI:** 10.1186/s13058-022-01583-3

**Published:** 2022-11-30

**Authors:** Juanjuan Gu, Redouane Ternifi, Soroosh Sabeti, Nicholas B. Larson, Jodi M. Carter, Robert T. Fazzio, Mostafa Fatemi, Azra Alizad

**Affiliations:** 1 Department of Physiology and Biomedical Engineering, Mayo Clinic College of Medicine and Science, Rochester, MN USA; 2 Department of Quantitative Health Sciences, Mayo Clinic College of Medicine and Science, Rochester, MN USA; 3 Department of Laboratory Medicine and Pathology, Mayo Clinic College of Medicine and Science, Rochester, MN USA; 4 Department of Radiology, Mayo Clinic College of Medicine and Science, 200 1St Street SW, Rochester, MN 55905 USA

**Keywords:** Contrast-free ultrasound, 3D microvessel imaging, Breast cancer, Vessel morphological features, Quantitative biomarkers

## Abstract

**Background:**

There is a strong correlation between the morphological features of new tumor vessels and malignancy. However, angiogenic heterogeneity necessitates 3D microvascular data of tumor microvessels for more reliable quantification. To provide more accurate information regarding vessel morphological features and improve breast lesion characterization, we introduced a quantitative 3D high-definition microvasculature imaging (q3D-HDMI) as a new easily applicable and robust tool to morphologically characterize microvasculature networks in breast tumors using a contrast-free ultrasound-based imaging approach.

**Methods:**

In this prospective study, from January 2020 through December 2021, a newly developed q3D-HDMI technique was evaluated on participants with ultrasound-identified suspicious breast lesions recommended for core needle biopsy. The morphological features of breast tumor microvessels were extracted from the q3D-HDMI. Leave-one-out cross-validation (LOOCV) was applied to test the combined diagnostic performance of multiple morphological parameters of breast tumor microvessels. Receiver operating characteristic (ROC) curves were used to evaluate the prediction performance of the generated pooled model.

**Results:**

Ninety-three participants (mean age 52 ± 17 years, 91 women) with 93 breast lesions were studied. The area under the ROC curve (AUC) generated with q3D-HDMI was 95.8% (95% CI 0.901–1.000), yielding a sensitivity of 91.7% and a specificity of 98.2%, that was significantly higher than the AUC generated with the q2D-HDMI (*p* = 0.02). When compared to q2D-HDMI, the tumor microvessel morphological parameters obtained from q3D-HDMI provides distinctive information that increases accuracy in differentiating breast tumors.

**Conclusions:**

The proposed quantitative volumetric imaging technique augments conventional breast ultrasound evaluation by increasing specificity in differentiating malignant from benign breast masses.

**Supplementary Information:**

The online version contains supplementary material available at 10.1186/s13058-022-01583-3.

## Background

For decades, angiogenesis has been recognized as a driving factor for rapid growth and metastasis of malignant solid tumors [1]; as such neovascularization plays a critical role in breast cancer growth and dissemination. Importantly, the structural abnormalities of malignant tumor microvessels are evident not only by high density but also by complexity of the newly formed vessels presenting with irregularity and tortuosity [2, 3]. Direct assessment of vessel morphological changes as biomarkers for cancer detection by imaging modalities is an emerging research interest.

Previous research has demonstrated the ability to obtain microvascular features of breast tumors at super-resolution scales [4] although no morphometric analysis was performed. A few studies have proposed ultrasound imaging of tumor microvessels for differentiation of breast masses without using contrast agents [5]; however, these efforts were limited to a pixel count method and visual inspection of images for the assessment of vessel shapes and distribution. Recently it has been demonstrated that slow blood flows can be successfully decoupled from strong tissue clutter signal when highly registered spatial–temporal data were processed in the singular-spectrum domain [6]. This approach includes a set of novel vessel enhancement processing algorithms to extract small vessels and suppress unrelated structures in the Power Doppler (PD) image. This approach provides tissue-blood echo separation and prepares the image for quantitative analysis of the microvasculature network and its morphology**.** Noting that this method can visualize small sub-millimeter vessels, as small as 300 µm, it has been termed high-definition microvasculature imaging (HDMI) [6, 7]. HDMI is primarily based on using ultrafast imaging, providing a significantly higher number of coherent imaging frames by utilizing plane-wave scanning instead of the conventional focused line-by-line scanning methods [6]. Quantitative analysis of microvessel morphological parameters as new biomarkers is described in [8]. In this work, morphological parameters of 2D HDMI images of breast lesions were extracted and used for lesion characterization [9]. In a more recent comparative study [10], it has been shown that adding the morphological parameter to those of shear wave elastography parameters improves the overall lesion characterization performance.

The integrity of the microvasculature morphology is vital for quantitative analysis [11]. As angiogenesis leads to formation of chaotic and tortuous vessels in malignant lesions [12], vascular information obtained from single imaging plane approach is incomplete [13]. Furthermore, 2D imaging methods overlook some important 3D structural features of microvessels and their connectivity, leading to underestimation or overestimation of different morphological parameters in a 2D plane [14, 15]. Therefore, 3D microvasculature imaging would be helpful for overcoming these limitations as more complete microvasculature information can be obtained.

3D microvasculature imaging for differentiation of breast masses including volumetric photoacoustic imaging, [16, 17] and 3D ultrasound localization microscopy [4, 11–13] are active research area; however, the latter requires IV placement, contrast medium injection and is costly. Efforts towards using contrast-free 3-D ultrasound imaging of microvessels, taking advantage of a high frame rate ultrasound technique, and providing volumetric information to differentiate breast masses using a vascular index have been reported [18]; however, morphometric analysis was not provided in that study.

In this work, we introduced a contrast-free quantitative three-dimensional high-definition microvasculature imaging (q3D-HDMI) method to provide microvasculature morphological information within a tumor volume. The q-HDMI methods (3D and 2D) objectively classify breast tumors in benign or malignant, which makes this method less operator dependent and eliminates the observer/reader variability providing a reliable clinical ultrasound imaging. Both the q2D-HDMI and q3D-HDMI techniques are not FDA cleared/approved and they remain investigational. We tested the hypothesis that the proposed contrast-free 3D microvasculature imaging provides complete and more accurate information regarding vessel morphological features and outperforms q2D-HDMI approach in distinguishing malignant from benign breast masses.

## Materials and methods

### Participants

**Fig. 1:**
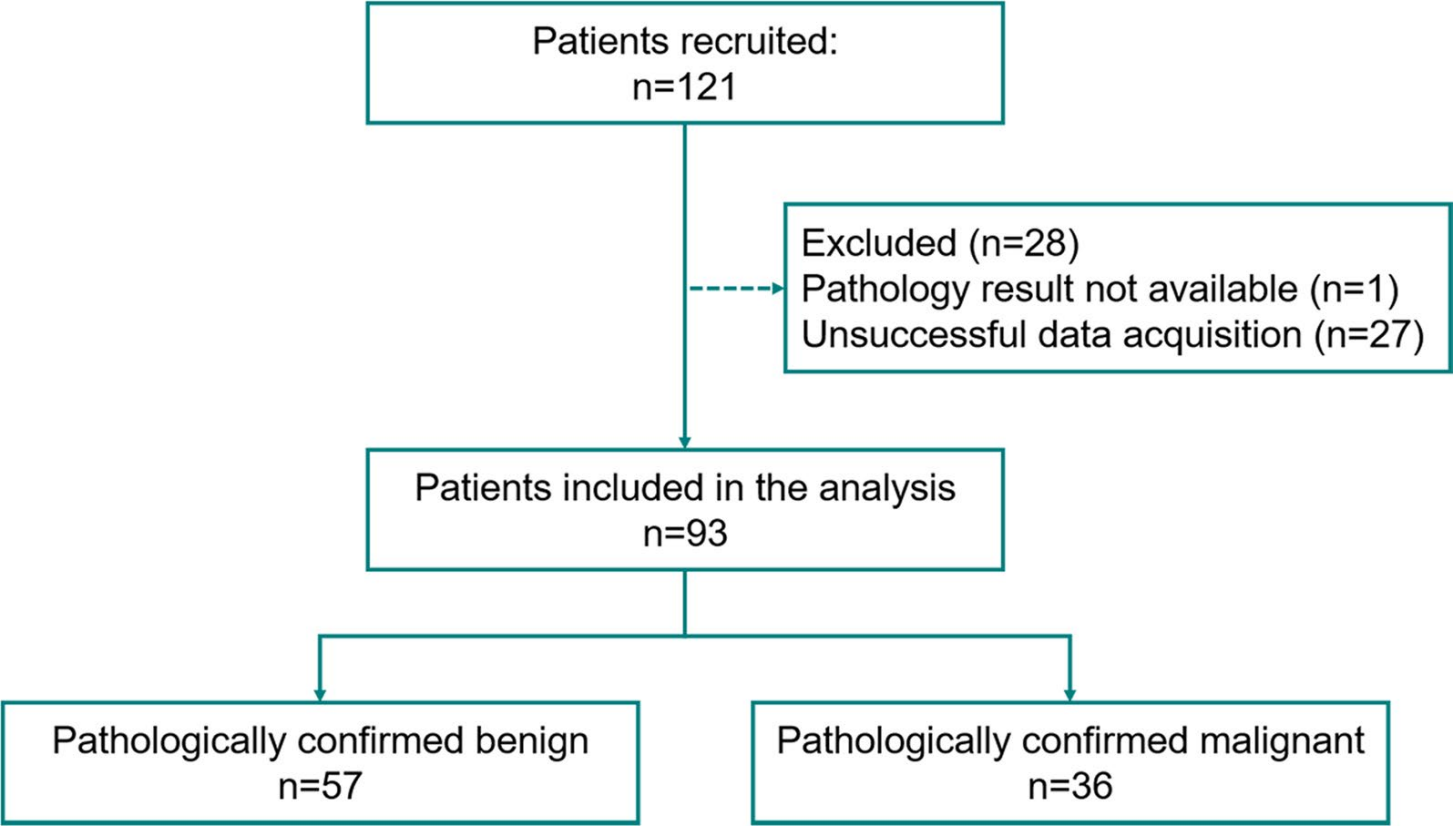
Flowchart for the participants enrolled in this study

This prospective study was approved by our institutional review board (IRB#: 19-003028) and was Health Insurance Portability and Accountability Act-compliant. Patients over 18 years old were referred by their primary physician to the department of Radiology for diagnostic breast imaging, including conventional ultrasound. One radiologist with 20 years of experience in breast imaging participated in this study. Moreover, the interpretation of clinical breast imaging, BI-RADS classification using grayscale ultrasound BI-RADS features as well as biopsy localization have been done by six different radiologists with 10–30 years of experience in breast imaging; however, they were not part of our investigative team and did not participate in data interpretation and or data analysis. Those patients with ultrasound-identified suspicious breast lesions recommended for biopsies were enrolled for this study. A signed written informed consent with permission for publication was obtained from each enrolled participant prior to the study. From January 2020 through December 2021, 2 males and 91 females were enrolled in this study. None of the patient population used in this study was previously reported. Participants’ information is summarized in Fig. 1. No selection bias, such as on age, sex and mass size or presence of vascularity in breast masses was present in our study eliminating pre-test probability of malignancy. All participants underwent core needle breast biopsy within an hour after the q3D-HDMI studies. Therefore, all the benign lesions were pathologically confirmed and did not need follow-up visits**.** A 14-gauge cutting needle was utilized. Five biopsy cores were obtained in each case. Pathology results reported by an experienced breast pathologist with more than 20 years’ experience served as the reference gold standard.

### Quantitative 3D high-definition microvasculature imaging

**Fig. 2:**
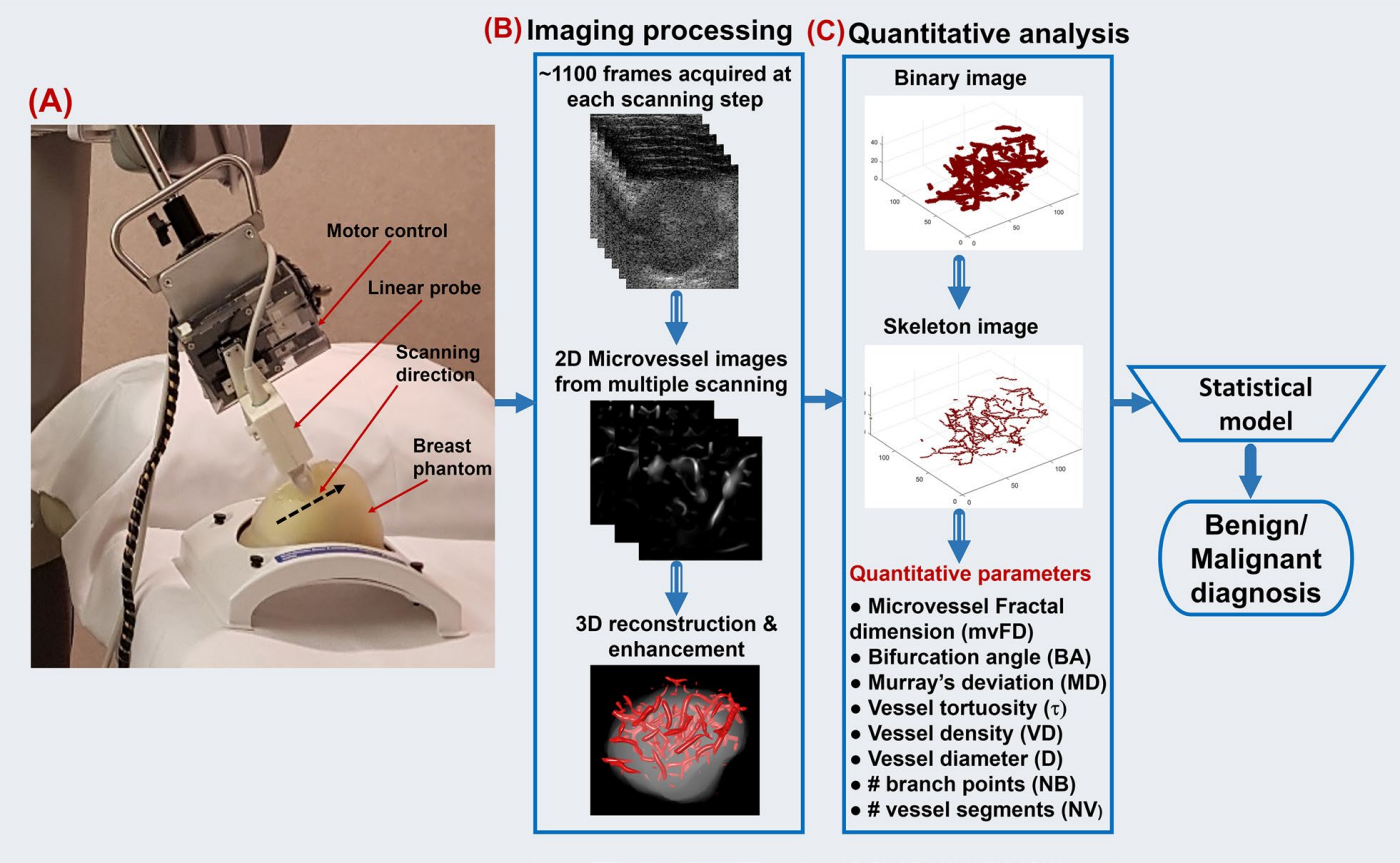
**a** Illustration of the 3D data acquisition process. The linear array moves along the black dotted arrow to acquire 3D data at a step size of 0.5 mm. **b** Images acquired at each scanning step were processed and rendered to generate the 3D microvessel structure. **c** Quantitative parameters were extracted based on the 3D structure. Finally, the quantitative parameters could be input to the statistical model for lesion differentiation. HDMI = high-definition microvasculature imaging

Ultrasound scanning was performed by one of two experienced sonographers with more than 30 and 15 years of clinical ultrasound scanning experience. A programmable ultrasound machine, Verasonics Vantage 256 system (Verasonics Inc., Redmond, WA), synchronized with a mechanical servo control scanning system was used for the study. A linear probe (L11-4v with central frequency at 6.25 MHz) was mounted on a motorized mechanical translation arm. The motor controller translated the linear array transducer along the elevational direction during data acquisition (Fig. 2a). Patients were asked to lie down on the back. If the lesion was located at the breast outer quadrant, a pillow was put behind the back with the patient face towards the side. It is known that peritumoral microvasculature and its morphology are important distinctive factors between malignant and benign [9, 19, 20]. Accordingly, our scanning method was designed to include the peritumoral vascularity. For this purpose, the sonographer first located the breast mass on the B-mode image and moved the probe to identify mass margins in all directions. Then, depending on the mass size, the sonographer repositioned the ultrasound probe to a few or several millimeters outside the mass margin into the normal background tissue. Starting from this point, the probe was set to automatically scan the breast in steps, covering the entire mass from one side to the other, and stopped a few to several millimeters after the mass margin has totally disappeared from the image on the opposite side. The sonographer monitored the whole scanning process to confirm that the whole mass was scanned properly. The translation step size was kept at 0.5 mm for all studies, providing a balance between acquisition time and 3D image quality. At each step, plane wave imaging was performed at a pulse repetition frequency of 550 Hz and 5 compounding angles. One thousand one hundred frames were acquired. The transducer was then placed on freeze mode for 2 s to save the IQ data. Next, the controller translated the transducer to the next scanning plane to continue data acquisition. Both the transverse and longitudinal orientations were acquired for most of the patients. For the lesions very close to the nipple, only one orientation easier for mechanical arm movement was scanned. In this study, the total scanning time was less than 5 min along each orientation. During data acquisition, the patient was instructed to remain still and breathe normally.

### 3D reconstruction and quantification of vessel morphology

For each lesion, the raw data acquired at each scanning step was first processed using MATLAB (MathWorks, Natick, MA) with the q2D-HDMI technique as detailed in [6, 7]. Briefly, clutter filtering, background noise reduction and a series of morphology filtering techniques were applied to extract the microvessels. This process resulted in a set of successive 2D B-mode images and the corresponding microvessel images of lesions along the scanning direction, as shown in Fig. 2b.

The 2D images were then imported into a commercial software package, Amira 3D (Thermo Fisher, Waltham, MA), for 3D reconstruction [21]. Details for the 3D volume rendering setup are shown in Appendix 1. Figure 2b shows an example with the reconstructed microvessel image overlaid on the dilated lesion image. Finally, the 3D structure was exported as a series of 2D images for extracting quantitative parameters. To include more peripheral zone vascularity, each extracted 2D image from 3D scanning was segmented with 2 to 5 mm dilation operation, depending on size of breast mass.

MATLAB was used for quantitative analysis of the 2D images obtained from Amira 3D. The thinning algorithm as described in [7, 22] was used to extract the skeleton. Figure 2c shows the 3D skeleton image example reconstructed using MATLAB. The skeleton image was used for extracting quantitative parameters: vessel density (VD), maximum vessel diameter (*D*_max_), mean vessel diameter (*D*_mean_), maximum Murray’s deviation (MD_max_), mean Murray’s deviation (MD_mean_), maximum bifurcation angle (BA_max_), mean bifurcation angle (BA_mean_), microvessel fractal dimension (mvFD), number of branch points (NB), number of vessel segments (NV), mean tortuosity (*τ*_mean_) and maximum tortuosity (*τ*_max_). The same parameters were also obtained for q2D-HDMI by analyzing the microvessel image acquired at the scanning step, which corresponded to the B-mode image with the largest lesion cross section. The detailed list of microvessel morphological Parameters is shown in Table 1.

**Table 1:** List of the quantitative microvessel morphological parameters

Biomarkers	Definition and calculation
NB	Numbers of branch points: defined as any node that is connected to three or more vessel segments
NV	Number of vessel segments
VD	Vessel density: defined as the proportion of vessel area with blood flow over the total area measured
*D* (mm)	Vessel diameter (*D*_mean_, *D*_max_): defined as two times of the minimum distance between the vessel centerline and the vessel border
τ	Vessel tortuosity (τ_mean_, τ_max_): Distance metric measures vascular tortuosity
MD	Murray’s deviation: diameter mismatch, defined as the deviation from Murray's law, increases in the vasculature network of malignant tumors
mvFD	Microvessel fractal dimension: A unit-less geometrical feature that is a marker of microvascular complexity
BA (°)	Bifurcation angle: refers to the angle between two daughter vessels

Note ‒ Subscript mean and max indicate the corresponding mean and maximum value, respectively

### Statistical analysis

Statistical analysis was conducted with RStudio (R version 4.0.4, Boston, MA, USA). The Wilcoxon rank sum test was used to compare the quantitative parameters by malignancy status. A two-sided *p* value less than 0.05 was considered statistically significant. In cases, where the vessel has no branches, the values for BA_mean_, BA_max_, MD_mean_ and MD_max_ parameters are undefined, and the quantification algorithm outputs a not a number (NaN) value. Analysis with the NaN value is described in Appendix 2. All quantitative parameters, including the sub-parameters were combined using the multi-variable logistic regression analysis, and those with high *p* values (> 0.05) were dropped from the model as the addition of these parameters did not improve the prediction accuracy. To finally test the combined diagnostic performance of multiple morphological parameters, including the sub-parameters, leave-one-out cross-validation (LOOCV) was used. The LOOCV method has the advantage that it can compensate for the overfitting problem due to a limited sample size [23]. Malignancy probability was calculated for each lesion, and $\mathrm{malignancy probability }={\mathrm{ logit}}^{-1}(B+\sum_{i=1}^{n}{C}_{i}{P}_{i})$, where $B$ and ${C}_{i}$ are constants obtained from LOOCV, ${C}_{i}$ is the coefficient for the corresponding quantitative biomarker ${P}_{i}$; $n$ is the number of quantitative biomarkers included in the prediction model; ${\mathrm{logit}}^{-1}(\alpha )\hspace{0.17em}=\hspace{0.17em}1/(1\hspace{0.17em}+\hspace{0.17em}\mathrm{exp}(-\alpha ))$. Receiver operating characteristic (ROC) curves were used to evaluate the performance of the pooled model predictions generated from the LOOCV analysis, and the optimal cut-point was defined as the point closest to the point (0,1) on the ROC curve. The corresponding 95% confidence interval (CI), sensitivity, specificity, positive predictive value (PPV) and negative predictive value (NPV) were compared to evaluate the performance of different models. DeLong's test for paired observations [24] was also conducted based on the area under the curve (AUC) for the model performance comparison. ROC curves generated with the VD, NB, *τ*_mean_, mvFD, MD_max_, MD_mean_, BA_max_ and BA_mean_ for differentiating benign from malignant lesions using q2D-HDMI and q3D-HDMI were compared. For both q2D-HDMI and q3D-HDMI, quantitative parameters VD, mvFD, *τ*_max_, NV, NB, *D*_mean_ were included in the LOOCV model.

## Results

### q3D-HDMI biomarkers outperformed q2D-HDMI biomarkers for breast lesion differentiation

Of the 93 enrolled patients (mean age 52 ± 17 years, ranges from 18 to 84 years, 2 men and 91 women), 93 breast masses were examined by q3D-HDMI imaging. Table 2 summarizes the lesion information included in this study. Lesion size along the largest dimension ranged from 3 to 46 mm with a mean value of 14.7 ± 9.4 mm. Ultrasound guided core needle breast biopsy results revealed 57 benign and 36 malignant lesions. The most common benign histologic type was fibroadenoma *n* = 24 (42.1%). Among the malignant lesions, the most common was invasive ductal carcinoma (IDC), *n* = 28 (77.8%). The morphological parameters of q3D- and q2D-HDMI for benign and malignant lesions are summarized in Table 3. For both the q2D-HDMI and q3D-HDMI, the values for VD, mvFD, NV, *τ*_max_ and *τ*_mean_ were significantly higher for malignant lesions with *p* < 0.001. Significant differences between benign and malignant lesions were found for both the *D*_mean_ and *D*_max_ obtained from q2D-HDMI with *p* < 0.001 and *p* = 0.002, respectively. However, no significant difference was found for *D*_mean_ or *D*_max_ obtained from q3D-HDMI with *p* = 0.35 and *p* = 0.42, respectively.

**Table 2:** Summary of the lesion information

	Lesion number
Total	93
Female	91
Male	2
BI-RADS 3	1
Benign	1
Malignant	0
BI-RADS 4	82
Benign	55
Malignant	27
BI-RADS 5	10
Benign	1
Malignant	9
Benign	57
Fibroadenoma	24
Fibrocystic changes and Dense stromal fibrosis	12
Pseudoangiomatous stromal hyperplasia (PASH)	3
^a^Atypical	3
Papillomas	4
Radial scar	3
^b^Other benign changes	8
Malignant	36
Invasive ductal carcinoma	28
Ductal carcinoma in situ	5
Invasive mammary carcinoma with mixed ductal and lobular features	3

^a^Atypical: atypical ductal hyperplasia cases (2) and atypical lobular hyperplasia case (1)

^b^Other benign changes: fat necrosis (1), duct ectasia (3), gynecomastia (1), cavernous hemangioma (1), tubular adenoma, organizing abscess with associated granulomatous reaction (1)

**Table 3:** q3D HDMI and q2D HDMI parameters for benign and malignant lesions

	Benign (57)	Malignant (36)	*p* value^a^
3D HDMI			
VD	0.001 ± 0.001	0.003 ± 0.002	< .001
* D*_mean_ (mm)	657.6 ± 99.2	637.5 ± 94.7	.35
* D*_max_ (mm)	1056.8 ± 242.6	1033.4 ± 240.5	.62
mvFD	0.932 ± 0.168	1.221 ± 0.161	< .001
NB	2.0 ± 4.9	13.5 ± 21.8	< .001
NV	7.1455 ± 11.5557	14.4 ± 12.2	< .001
* τ*_mean_	1.2 ± 0.2	2.0 ± 1.0	< .001
* τ*_max_	2.0 ± 1.5	8.6 ± 12.5	< .001
2D HDMI			
VD	0.0031 ± 0.0042	0.0177 ± 0.0372	< .001
*D*_mean_ (mm)	470.2 ± 529.8	731.4 ± 172.0	< .001
*D*_max_ (mm)	885.8 ± 1768.3	1118.1 ± 302.8	.002
mvFD	0.411 ± 0.409	0.872 ± 0.215	< .001
NB	0.018 ± 0.135	0.229 ± 0.490	.003
NV	1.7 ± 1.8	3.7 ± 2.9	< .001
* τ*_mean_	1.0 ± 0.03	1.1 ± 0.04	< .001
* τ*_max_	1.2 ± 0.2	1.4 ± 0.4	< .001

Data are presented as mean ± SD format

^a^p values are based on Wilcoxon rank sum test and a value less than .05 was considered statistically significant

HDMI = high-definition microvasculature imaging

**Fig. 3:**
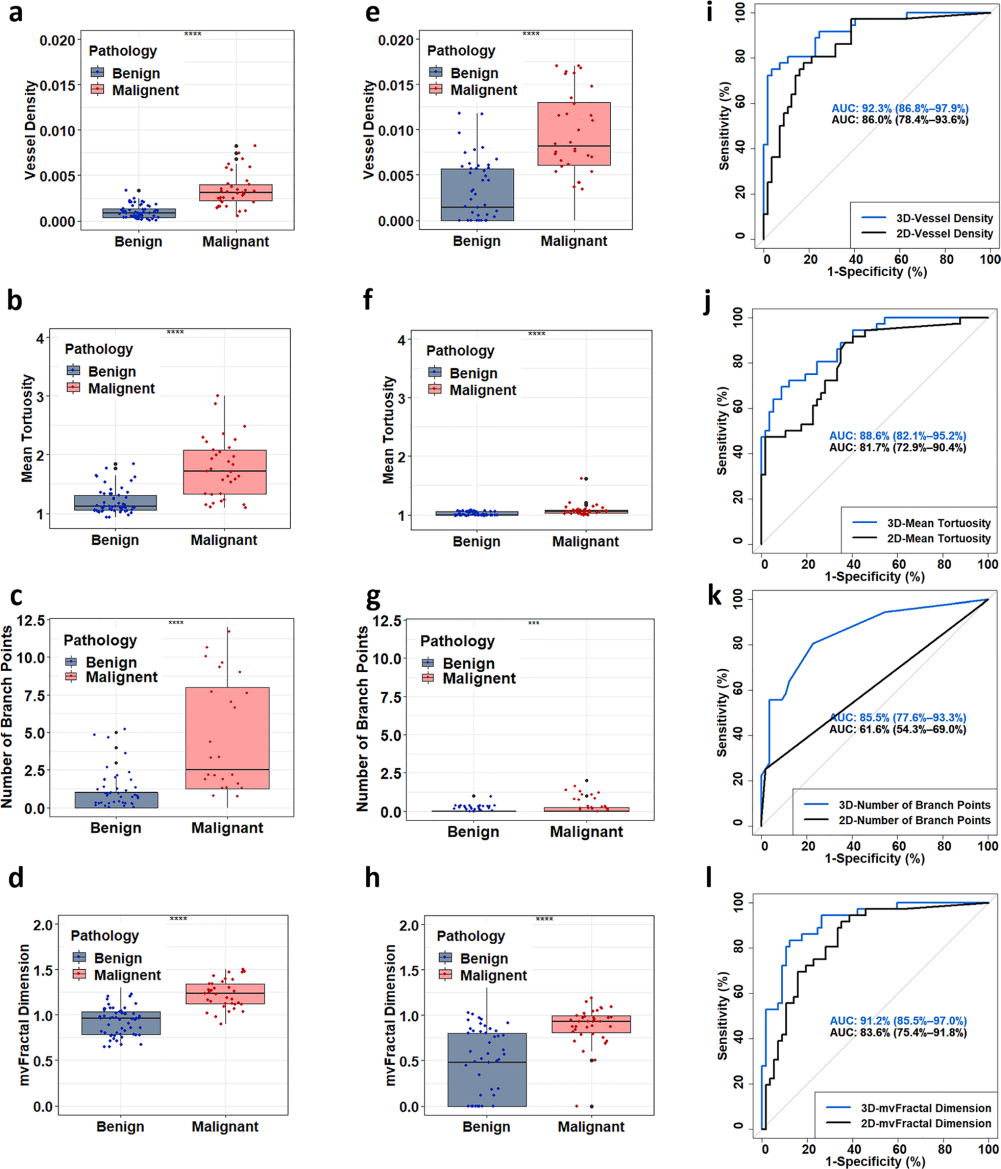
Comparison of the quantitative parameters between benign and malignant lesions obtained with q2D- and q3D-HDMI and the ROC curves. Parameters of q3D-HDMI are shown in first column: **a** vessel density, **b** number of branches, **c** mean tortuosity, and **d** microvessel fractal dimension. Parameters of q2D HDMI are shown in second column: **e** vessel density, **f** number of branches, **g** mean tortuosity, and **h** microvessel fractal dimension. Comparison of the ROC curves between q2D HDMI and q3D HDMI generated with **i** vessel density, **j** number of vessel segments, **k** mean tortuosity, and **l** microvessel fractal dimension. HDMI = high-definition microvasculature imaging

The boxplots for four representative biomarkers VD, NB, *τ*_mean_ and mvFD obtained with q3D-HDMI and q2D-HDMI are shown Fig. 3. Compared to q2D-HDMI, the q3D-HDMI showed higher mean NB, *τ*_mean_ and mvFD. Also, q3D-HDMI showed lower averaged VD values for both benign and malignant lesions; this was expected as the vessel density in 2D is calculated per unit area, whereas in 3D it is calculated per unit volume. As shown in Fig. 3, ROC curves generated with a parameter obtained from q3D-HDMI demonstrated improved discrimination versus those generated with the same parameter obtained from q2D-HDMI. Significant difference was found for the ROC curve for NB with the corresponding DeLong test *p* values < 0.001.

**Fig. 4:**
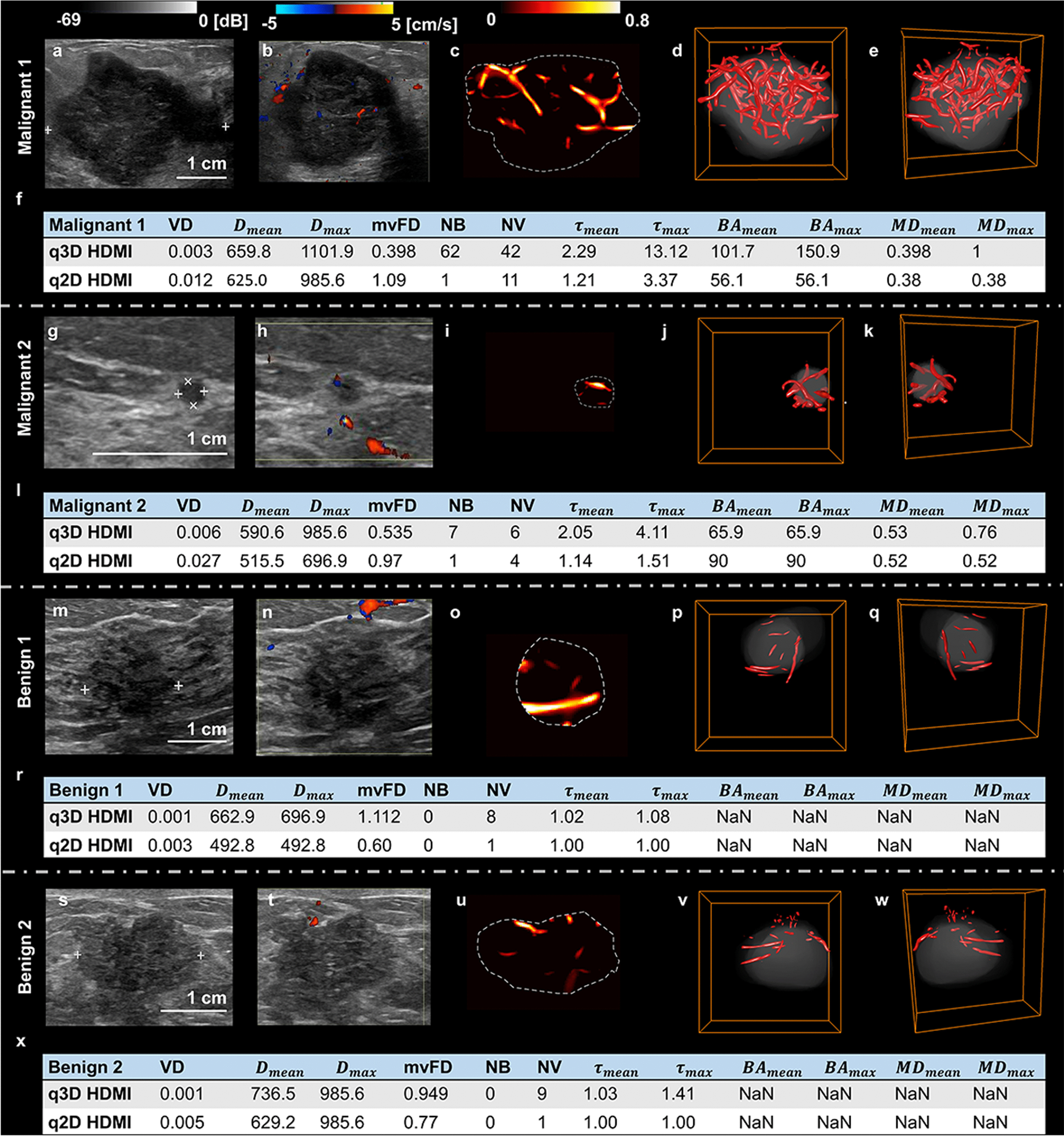
Visual presentation of conventional B-mode and Doppler ultrasound, q2D- and q3D-HDMI images of malignant and benign breast lesions. **a–f** Illustration of a 36 mm breast mass, invasive ductal carcinoma, Nottingham grade III, from a 55-yrs-old female. **g–l** Illustration of a small 3 mm breast lesion, invasive ductal carcinoma Nottingham grade II, from a 40-yr-old female. **m–r** Illustration of a 16 mm breast lesion, benign dense stromal fibrosis, from a 43-yrs-old female. **s–x** Illustration of a 26 mm mass due with pathology indicating benign gynecomastia from a 79-yrs-old male. The first column (**a, g, m, s**) are the clinical B-mode images. The second column (**b, h, n, t**) are the corresponding 2D color Doppler images. The third column (**c, i, o, u**) are the corresponding 2D high-definition microvasculature imaging (HDMI) images. The fourth (**d, j, p, v**) and the fifth (**e, k, q, w**) columns are the corresponding 3D HDMI images obtained at different views. The quantitative parameters for the two malignant and benign lesions obtained from q3D-HDMI and q2D-HDMI are shown in the figures (**f, l, r, x**). HDMI = high-definition microvasculature imaging

The conventional B-mode and Doppler ultrasound, q2D- and q3D-HDMI images of two malignant lesions, large (as large as 36-mm) and small (as small as 3-mm) and two benign breast lesions are shown in Fig. 4 for visual comparison. The corresponding videos of the 3D rendered microvasculature images are shown in Additional files 1–4. For both the large and small malignant lesions, while 2D-HDMI shows high-definition images of tumor vascularity, the 3D-HDMI shows complete connectivity of vascularity inside and around the breast tumor. For all lesions, there are more microvessels with complete connectivity in the q3D-HDMI images. Compared to q2D-HDMI, the benign lesion vessels shown in q3D-HDMI are more regular and straight with fewer branch points, presenting less complexity when compared to those of both of large and small malignant masses, indicating the benignity of these breast lesions. The quantitative biomarkers obtained by both q3D-HDMI and q3D-HDMI are detailed in the table in row below of each breast lesion in Fig. 4 for comparison.

### Lesion differentiation analysis with LOOCV

**Fig. 5:**
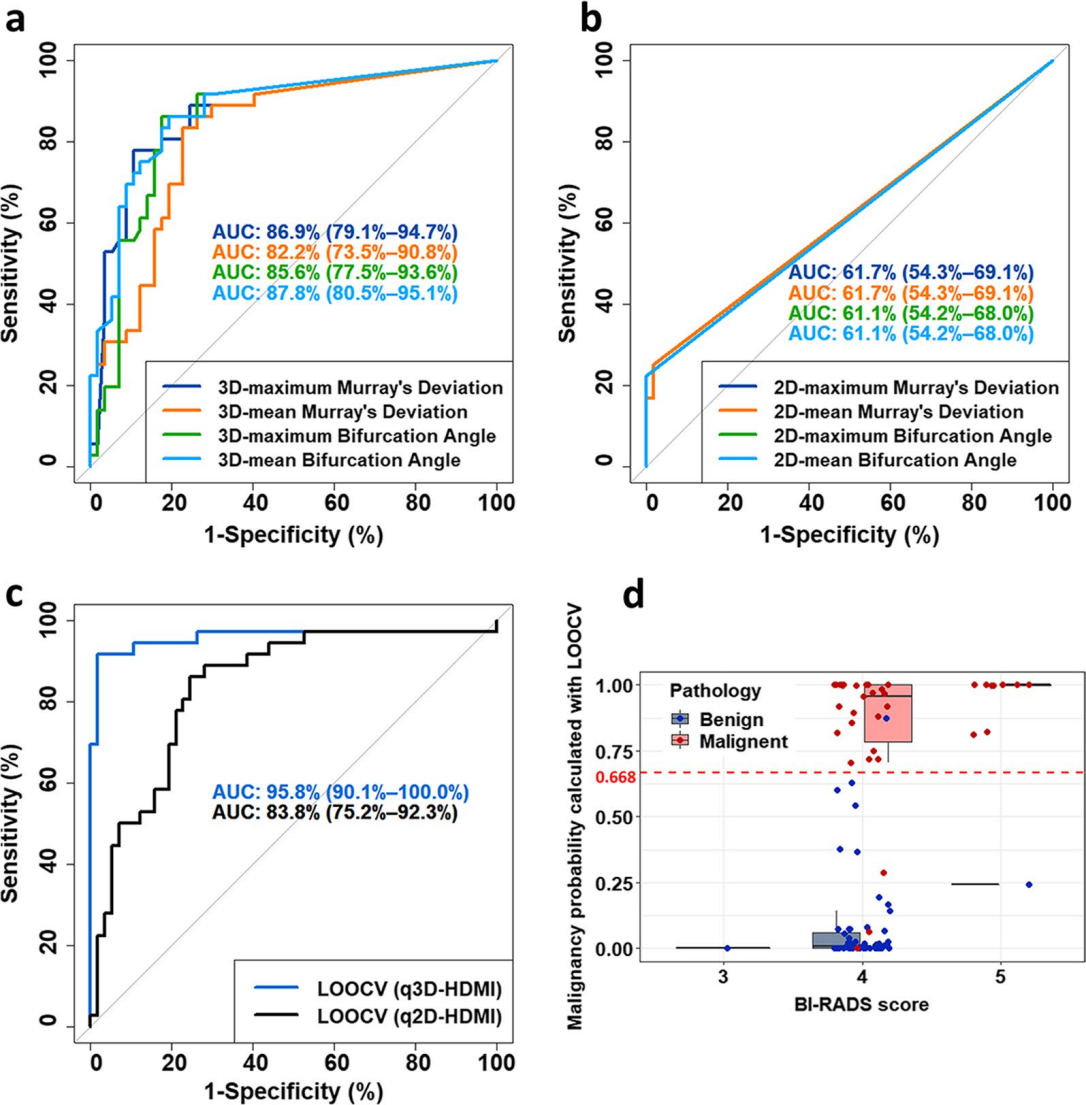
**a** ROC curves generated with MD_max_, MD_mean_, BA_max_ and BA_mean_ obtained from 3D high-definition microvasculature imaging (q3D-HDMI). **b** ROC curves generated with MD_max_, MD_mean_, BA_max_ and BA_mean_ obtained from 2D high-definition microvasculature imaging (q2D-HDMI). **c** ROC curves generated with the leave-one-out cross-validation (LOOCV) method for q2D-HDMI and q3D-HDMI method. For both q2D-HDMI and q3D-HDMI, quantitative parameters VD, mvFD, *τ*_max_, NV, NB, D_mean_ were included in the LOOCV model. **d** Boxplot of the distribution of the malignancy probability for each lesion in each BI-RADS category. The coefficients included in the equation for calculating the malignancy probability were obtained with LOOCV. The malignancy probability was calculated with LOOCV for the q3D HDMI. The dashed red line indicates the cutoff for differentiating benign from malignant lesions. The red dots represent the malignant lesions, and the blue dots represent the benign lesions. HDMI = high-definition microvasculature imaging. ROC = receiver operating characteristic. LOOCV = Leave-one-out cross-validation

The ROC curves generated with MD_max_, MD_mean_, BA_max_ and BA_mean_ obtained from q3D-HDMI and q2D-HDMI are shown in Fig. 5a and b, respectively. All four parameters obtained from q3D-HDMI gave a significantly higher AUC when compared to the results obtained from q2D-HDMI, with *p* < 0.001. For both q2D-HDMI and q3D-HDMI, quantitative parameters VD, mvFD, *τ*_max_, NV, NB, *D*_mean_ were included in the ROC Curves generated with the LOOCV model, as shown in Fig. 5c. The AUC for ROC curve of the q2D-HDMI was 83.8% (95% CI 75.2–92.3%). The corresponding sensitivity, specificity, PPV, and NPV were 86.1%, 75.4%, 68.9% and 89.6%, respectively. The AUC increased significantly (*p* = 0.02) for q3D-HDMI and it was 95.8% (95% CI 0.901–1.000). The corresponding sensitivity, specificity, PPV, and NPV were 91.7%, 98.2%, 97.1%, 94.9%, respectively. The negative likelihood ratio (NRL) for q2D-HDMI and q3D-HDMI were 0.184 and 0.085, respectively.

**Fig. 6:**
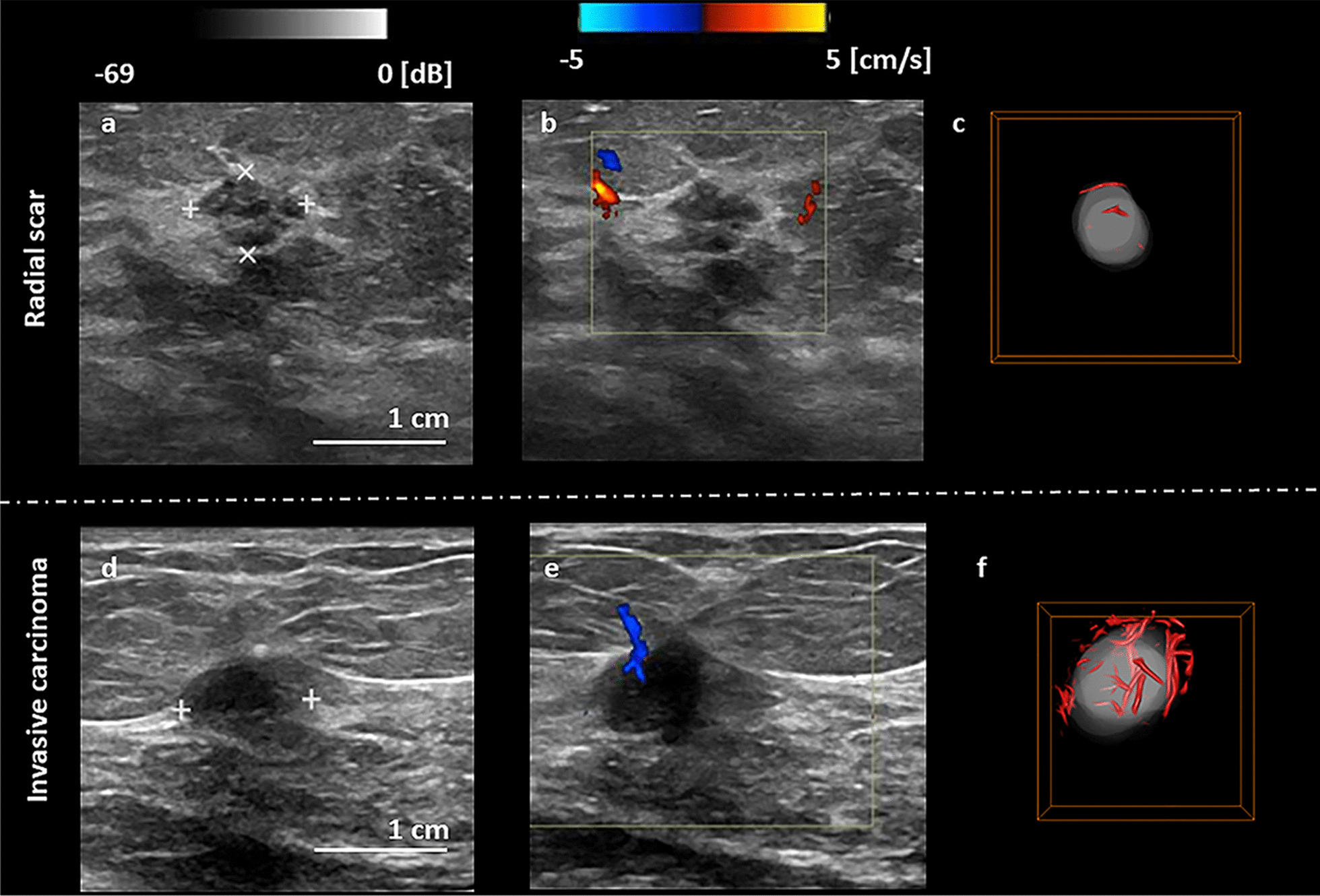
Visual presentation of conventional B-mode and Doppler ultrasound, and q3D-HDMI images of a radial scar and an invasive ductal carcinoma. **a-c** Illustration of a 8 mm breast mass, fragments of radial scar with florid usual type ductal hyperplasia and apocrine, from a 55-yrs-old female. **d–f** Illustration of a 10 mm breast mass, invasive ductal carcinoma, Nottingham grade II, from a 60-yrs-old female

The malignancy probability [9] was calculated with LOOCV for q3D-HDMI. Figure 5d shows the malignancy probability distribution for each lesion in each BI-RADS category for q3D-HDMI. The cutoff value of the malignancy probability is 0.668, denoted by a red dashed line in Fig. 5d. As illustrated in Fig. 5d, the benign and malignant lesions in BI-RADS 3 and 4 groups were captured successfully with the q3D-HDMI. There were three false negatives (represented by red dots below the red dashed line) and one false positive (represented by blue dot above the red dashed line). Among the three false negatives, there were two ductal carcinomas in situ (DCIS), low nuclear grade cribriform type, one 9 mm, and the second was 14 mm in diameter. The third false negative case was a male patient whose breast pathology indicating a 20 mm invasive ductal carcinoma, Nottingham grade II (of III), subtype category of Luminal A (estrogen and progesterone receptors positive, HER2- negative and low levels of the protein Ki-67). The pathology of false positive was a 12 mm size fibroadenoma with lactational changes in background breast parenchyma from a female patient. The biopsy results of benign lesions also revealed 3 radial scars and 12 fibrocystic changes without hyperplasia. The prediction model of q3D-HDMI correctly predicted all three radial scars and 12 fibrocystic changes as benign. Figure 6 shows an example of a 8 mm radial scar next to a 10 mm invasive ductal carcinoma.

## Discussion

In this study, we presented in vivo imaging of breast tumor microvessels volumetrically along with comprehensive quantitative analysis of tumor microvessels extracted from the novel q3D-HDMI for the first time. No contrast agent was applied. We demonstrated that q3D-HDMI outperforms the 2D approach and increases the accuracy in malignancy prediction of breast lesions.

The results of our study included one false positive. The pathology confirmed the lesion to be a fibroadenoma with lactational changes in background breast parenchyma. It is known that fibroadenoma is a hormone sensitive tumor and often becomes enlarged with increased vascularity during pregnancy and lactation. Furthermore, fibroadenoma tends to get atypical radiological features during pregnancy and lactation, necessitating core needle biopsy to confirm the diagnosis [25]. The study also included 3 false negatives. The pathology of two of false negatives was DCIS with low nuclear grade cribriform type. Studies show that low-grade DCIS tend to have less vascularity and may develop into low-grade invasive carcinoma [26]. Indeed, Grade 1 DCIS generates much less angiogenesis than high nuclear grade of DCIS [27]. In other words, the false negativity of these low-grade DCIS in our study is more physiologic than a technical issue. The third false negative was IDC Nottingham grade II (of III), subtype category of Luminal A. It is known that luminal A subtypes are slow growing cancers and have low microvessel density with less structural complexity and they maintain the best prognosis among all subtypes [28]. In other words, this false negative IDC was more physiologic than a technical issue.

Radial scars are often difficult to differentiate from IDC on conventional imaging and pathology review is often non-trivial [29]. All three radial scars included in this study were correctly predicted as benign. Thus, with q3D-HDMI, the microvasculature features of radial scars could assist for more accurate differentiation. Furthermore, the proposed prediction model correctly predicted all the masses with fibrocystic breast changes without evidence of hyperplasia as benign. Our finding is supported by report of previous study indicating that fibrocystic breast disease without hyperplasia shows lower grade microvessel density compared to those associated with hyperplasia [30].

Though, 3D power Doppler ultrasound for breast cancer diagnosis [31] and contrast agent mediated 3D ultrasound localization microscopy on tumor bearing rats [13] demonstrated the potential of such volumetric techniques; our study demonstrated the effectiveness of the q3D-HDMI through a comprehensive quantitative in vivo study for characterization of breast masses in human.

It is known that angiogenesis, the formation of new microvessels toward and within a malignant breast tumor, starts when the tumor reaches the size of 2–4 mm in diameter [32]. One of the important findings in this study is that q3D-HDMI was able to capture the microvasculature structures in a breast lesion as small as 3 mm. Furthermore, the q3D-HDMI displayed more vessels with better connectivity in this small tumor than what was possible with q2D-HDMI. As a result, the prediction model based on q3D-HDMI quantitative biomarkers correctly diagnosed this small lesion as malignant. It is known that male breast cancer has even more intense angiogenic reaction than female breast cancer [33], this is in support of our study, which included two men with breast masses and q3D-HDMI quantitative biomarkers correctly diagnosed them as invasive ductal carcinoma.

To the best of our knowledge, this study is the first to compare 3D and 2D microvessel images quantitatively and demonstrates that the new 3D microvasculature imaging technique, q3D-HDMI, is superior to the current q2D-HDMI method for several reasons. With 2D microvasculature imaging, only one plane of the lesion can be evaluated [13]. In our study, for the 2D analysis, the microvessel image corresponding to the B-mode image with the largest lesion area was selected. However, the microvasculature information in the malignant lesion is highly spatially heterogeneous, and therefore the microvessel information could largely vary from slice to slice, making it hard to select a representative 2D slice for analysis. Therefore, the microvasculature morphological features obtained on a single plane in 2D imaging may not be a true estimation and could be underestimated or overestimated [14]. For example, our study showed that the mvFD, NB and tortuosity information obtained from q3D-HDMI was significantly higher than the same parameters obtained from q2D-HDMI and significantly different in benign and malignant. In addition, q2D-HDMI can visualize the vessel only in a two-dimensional space, hence vessels extending outside the imaging plane and their morphological parameters would not be captured. On the other hand, q3D-HDMI captures the entire vessel network and their morphological parameters, providing a more accurate estimation of such parameters. This finding is in agreement with previous study [14]. Our study also showed that the vessel density measured with q2D-HDMI was significantly higher than that measured with 3D, indicating that q2D-HDMI overestimated microvessel density. However, this is expected as the vessel density in 2D is calculated per unit area, whereas in 3D it is calculated per unit volume.

In this study, the recently introduced parameters for breast cancer detection, Murray’s deviation [8, 34], representing the relationship between mother vessel and daughter vessels and bifurcation angle [8, 35] based on information between the daughter vessels, were significantly different in benign and malignant in q3D-HDMI approach, indicating its ability to accurately visualize branches information in a 3-dimensional space. However, for q2D-HDMI, most of the lesions resulted in NaN values for these two parameters, suggesting that no daughter vessels were observed.

Microvessel morphology and its distribution feature vary between benign and malignant breast tumors and are likely to be an important discrimination marker [2, 36]. Studies have shown that morphological parameters of tumor microvessels obtained from contrast-free quantitative 2D-HDMI increases the sensitivity and specificity in discriminating malignant from benign breast masses [7, 9, 10]. The study presented here shows that morphological biomarkers of microvasculature network obtained by 3D-HDMI outperforms those of q2D-HDMI and increases the sensitivity and specificity in differentiating malignant from benign breast masses. It is known that the morphology of the vasculature in the immediate vicinity of breast tumors plays a significant role in differentiation between cancerous and benign masses. Studies in [19, 37] used a combined optoacoustic and ultrasound images of breast tumors to show that aggressively growing malignant tumors tend to recruit vasculature from the immediate proximity so that small arteries penetrate cancerous tumors radially. In contrast, benign tumors push surrounding vasculature out, so those vessels appear on and parallel to the surface of fibroadenomas.

The performance of combined OA and grayscale US for breast lesion differentiation has been shown in [19, 37]. Study in [19] also showed that OA/US increased the specificity of breast mass assessment compared with the device internal grayscale ultrasound alone. A direct comparison of the sensitivity/specificity for HDMI and OA/US requires a separate comparative study. However, since HDMI and OA/US present different characteristics of breast lesions, it is expected that they could complement each other in breast lesion differentiation. Therefore, it will be interesting to combine q3D-HDMI with OA/US in the future to further improve the specificity of breast lesion differentiation and reduce the unnecessary biopsies.

Our study had limitations. The sample size was small. Also, to have pathology as gold reference standard, only the participants with recommendation for breast biopsy, nearly all BI-RADS 4 and 5, were enrolled in this study. This prevented a direct comparison between q3D-HDMI and gray scale ultrasound. In the future, we plan to apply this method on a larger population irrespective of their BI-RADS category. Also, there is a potential for data degradation due to breathing motions, as patients were allowed to breathe normally during data collection. In the future we plan to utilize and expand the motion correction algorithms [38–40] to reduce potential motion artifacts or to use deep learning technique [41, 42] with potential for correction of motion artifacts.

## Conclusions

In summary, our study comprehensively assesses tumor microvessel morphological features in a three-dimensional space using our novel contrast-free ultrasound-based quantitative 3D-HDMI. All quantitative parameters obtained from the q3D-HDMI outperformed the q2D-HDMI in differentiating malignant versus benign breast masses. Appendix 3 explains a practical way for applying this new q3D-HDMI technique clinically. In the future, the proposed method can offer a new means for breast mass characterization when used as a complementary imaging tool to conventional ultrasound.

### Supplementary Information

**Additional file 1.** 3D video of the rendered 3D microvasculature image for a 36 mm invasive ductal carcinoma from a 55-yrs-old female with Nottingham grade III.

**Additional file 2.** 3D video of the rendered 3D microvasculature image for a 3 mm invasive ductal carcinoma from a 40-yrs-old female with Nottingham grade II.

**Additional file 3.** 3D video of the rendered 3D microvasculature image for a 16 mm *benign dense stromal fibrosis* from a 43-yrs-old female.

**Additional file 4.** 3D video of the rendered 3D microvasculature image for a 26 mm benign *Gynecomastia* from a 79-yrs-old male.

## Figures and Tables

**Fig. 7:**
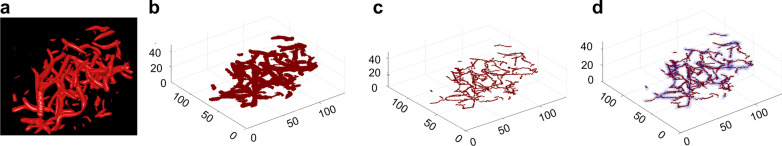
Set of q3D-HDMI processing steps after image formation and segmentation to prepare the image for quantification. **a** Microvasculature image of malignant breast tumor obtained from Amira 3D, **b** binary microvasculature image generated in MATLAB, **c** skeleton showing the network of vessels, **d** the skeleton overlaid on the binary vessel image. HDMI = high-definition microvasculature imaging

## Data Availability

The data that support the findings of this study are available from the corresponding author upon reasonable request. The requested data may include figures that have associated raw data. Because the study was conducted on human volunteers, the release of patient data may be restricted by Mayo policy and needs special request. The request can be sent to: Karen A. Hartman, MSN, CHRC | Administrator—Research Compliance| Integrity and Compliance Office | Assistant Professor of Health Care Administration, Mayo Clinic College of Medicine & Science | 507-538-5238 | Administrative Assistant: 507-266-6286 | hartman.karen@mayo.edu Mayo Clinic | 200 First Street SW | Rochester, MN 55905 | mayoclinic.org. We do not have publicly available Accession codes, unique identifiers, or web links.

## Appendix 1: 3D volume rendering using Amira 3D and quantification

After importing the 2D images to the Amira 3D software, the voxel size along the elevational direction was set as 0.25 mm as the scanning step size was 0.5 mm. The B-mode images at different slices were segmented along the lesion boundary to reconstruct the 3D volume of the lesion. Similarly, the 3D microvessel image was reconstructed using the ensemble of the 2D microvessel images along the elevational direction. The “Structure enhancement filter” function was first applied to automatically identify the blood vessels, and therefore, enhanced the 3D structures [21]. With this filter, a score between 0 and 1 was computed for each pixel in the 3D image, with 0 representing the background and 1 representing a good matching with the structure model. The score was calculated based on the Hessian matrix of the image and the Hessian matrix was computed by filtering the image with the derivative of a Gaussian kernel. The size of the Gaussian kernel controlled the scale of analysis since the structures were detected from minimum standard deviation value to the maximum value at a constant step size. In this study, the standard deviation maximum and minimum values were set as 1 and 2, respectively, and the step was set as 0.5. The lesion volume was dilated 2 mm along the segmented lesion boundary, and the 3D microvessel image was masked with the dilated lesion volume.

Later, the “Threshold by criterion” function was applied to the 3D structure. The criterion value was set between 0 and 1.0, and the signals with amplitude less than the criterion value were considered as artifacts or noise and were removed. For most of the lesions, the threshold was set empirically as 0.03. A smaller threshold was applied for deep-seated lesions as it was expected that signal intensity decreased with increased penetration [11, 43]. A larger threshold was applied if obvious large motion artifacts existed. Appendix Fig. 7a shows an example for the 3D microvessel structure. Next, the “Fill holes” function was used to fill the holes inside the 3D volume structure using the interpolation method in which voxels with at least one common vertex were considered connected, irrespective of the volume structure size. After that, the “Remove small spots” function was used to remove 3D objects with total size less than 30 pixels as they were considered as noise or artifacts.

MATLAB was used for quantitative analysis of the 2D images obtained from Amira 3D. Appendix Fig. 7b shows the 3D microvessel structure generated using MATLAB. The thinning algorithm as described in [7, 44] was used to extract the skeleton. Appendix Fig. 7c shows the binary skeleton image. The skeleton image was used for extracting quantitative parameters, as described in [7, 8].

## Appendix 2: Statistical analysis with the parameters with NaN value

In cases, where the vessel has no branches, the values for BA_mean_, BA_max_, MD_mean_ and MD_max_ parameters are undefined, and outputs a not a number (NaN) value. These NaN values embody structural missingness and contain information that either no vessel was detected in the lesion, or if there was a vessel, it did not have any branches. Therefore, it is important to use this information in the statistical analysis. Two sub-parameters were derived for each of the above-mentioned parameters. The first sub-parameter was assigned a binary value of 0 if the parameter value was a NaN, or 1, if the parameter value was not a NaN. With this definition, this sub-parameter simply indicates the presence or absence of a branch. The second sub-parameter was defined as a continuous variable; it was assigned 0 if the parameter value was a NaN, or the original value of the parameter if it had a numeric value. In order to evaluate the diagnostic performance of individual parameters BA_mean_, BA_max_, MD_mean_ and MD_max_, the pair of sub-parameters corresponding to each of these parameters were tested separately using multivariable logistic regression analysis.

## Appendix 3: Clinical application of the quantitative 3D high-definition microvasculature imaging

The q3D-HDMI is a quantitative approach, and objectively classifies breast tumor in benign or malignant, which makes this method less operator dependent and eliminates the observer/reader variability. The clinical application of this technique would be: 1) quantitative biomarkers, which are extracted automatically by the ultrasound machine implemented with the q3D-HDMI algorithm, are combined with the prediction model and a malignancy probability will be given for each scanned lesion. The scanning time required for each lesion along each orientation is less than 5 min. With advanced and powerful computation system, real time processing is possible. 2) The malignancy probability is then compared with the cutoff value. The cutoff value proposed in this study does not represent the final optimized value for general population. Multi-center, large population studies should be conducted to calculate and validate the cutoff value. 3) If the malignancy probability is higher than the cutoff, indicating that the 3D scanning supports the biopsy. Otherwise, the 3D scanning supports for follow-ups. We want to clarify that the q3D-HDMI technique proposed in this study is not used for replacing the conventional B-mode, or current imaging techniques, but as a complementary for improving the sensitivity and sensitivity in breast lesion differentiation. The final decision whether the patient should undergo the biopsy should be the decision of the radiologist.

## Abbreviations

qHDMI: Quantitative high-definition microvasculature imaging
ROC: Receiver operating characteristic
LOOCV: Leave-one-out cross-validation
AUC: Area under the curve
NaN: Not a number
CI: Confidence interval
PPV: Positive predictive value
NPV: Negative predictive value
IDC: Invasive ductal carcinoma

## Code availability

The custom code or mathematical algorithms that are deemed central to the conclusions are available from the corresponding author upon request.
